# Adaptive Neuroplastic Responses in Early and Late Hemispherectomized Monkeys

**DOI:** 10.1155/2012/852423

**Published:** 2012-06-27

**Authors:** Mark W. Burke, Ron Kupers, Maurice Ptito

**Affiliations:** ^1^Department of Physiology and Biophysics, College of Medicine, Howard University, Washington, DC 20059, USA; ^2^Institute of Neuroscience and Pharmacology, University of Copenhagen, 2200 Copenhagen, Denmark; ^3^School of Optometry, University of Montreal, Montreal, QC, Canada H3C 3J7

## Abstract

Behavioural recovery in children who undergo medically required hemispherectomy showcase the remarkable ability of the cerebral cortex to adapt and reorganize following insult early in life. Case study data suggest that lesions sustained early in childhood lead to better recovery compared to those that occur later in life. In these children, it is possible that neural reorganization had begun prior to surgery but was masked by the dysfunctional hemisphere. The degree of neural reorganization has been difficult to study systematically in human infants. Here we present a 20-year culmination of data on our nonhuman primate model (*Chlorocebus sabeus*) of early-life hemispherectomy in which behavioral recovery is interpreted in light of plastic processes that lead to the anatomical reorganization of the early-damaged brain. The model presented here suggests that significant functional recovery occurs after the removal of one hemisphere in monkeys with no preexisting neurological dysfunctions. Human and primate studies suggest a critical role for subcortical and brainstem structures as well as corticospinal tracts in the neuroanatomical reorganization which result in the remarkable behavioral recovery following hemispherectomy. The non-human primate model presented here offers a unique opportunity for studying the behavioral and functional neuroanatomical reorganization that underlies developmental plasticity.

## 1. Introduction

### 1.1. Prologue

Cerebral hemicorticectomy is a form of radical surgical intervention currently used in the treatment of intractable epilepsy [1] and malignant tumors [2] accompanied by infantile hemiplegia [3, 4].Neurological and behavioral functions are remarkably improved following the removal of the entire cerebral hemisphere, not only in infants but also in adults, with the recovery being greater for the early-lesioned subjects [5, 6]. Although hemispherectomized patients may go on to lead full lives, it is not complete and individuals have lingering behavioral manifestations [7].

The degree of recovery largely depends on the system being investigated. For example, motor functions are improved postoperatively, locomotion is preserved, and the hemiplegia of the contralateral limb is ameliorated with the apparition of simple voluntary movements [4]. Thresholds for touch, pain and temperature are elevated [4, 8, 9], and localization and discriminative abilities are diminished. Sensory functions are better preserved for the face and the leg and are worsened for the forearm and the hand [4, 9]. At the visual level, there is a persistent contralateral hemianopia similar to the one observed following massive damage to the primary visual area. In clinical settings, functional reorganization may be masked by the dysfunctional hemisphere, providing the illusion of a rapid behavioral recovery [10, 11].

In recent years, we have developed a primate model of human hemispherectomy that allowed us to study behavioral recovery and its underlying anatomical substrates [12, 13]. This model eliminates the potential contamination of residual cortical areas and their projections that can participate in the reorganization process, as is the case in studies using discrete lesions as well as potential presurgery reorganization [10]. Furthermore, by sparing subcortical structures such as striatum, diencephalon, and brainstem, this model provides insights into the mechanisms involved in the magnitude of behavioral recovery.We summarize in this paper results on longitudinal behavioral assessment of sensory and motor functions as well as a histological overview of brain reorganization with an emphasis on potential neural substrates for behavioral recovery.

### 1.2. General Experimental Procedure

#### 1.2.1. Subjects

Eight infants (median age of 9 weeks) and two adult (48 months of age) Vervet monkeys (*Chlorocebus sabeus*) were used in these studies and underwent the removal of the entire left cerebral hemisphere. An additional 2 adult monkeys without surgical procedures were used as normal controls. All subjects were housed in an enriched naturalistic environment at the facilities of the Behavioral Sciences Foundation, St Kitts as previously described [14]. The experimental protocol was reviewed and approved by the University of Montreal Animal Care and Use Committee.

#### 1.2.2. Surgery

**Figure 1:**
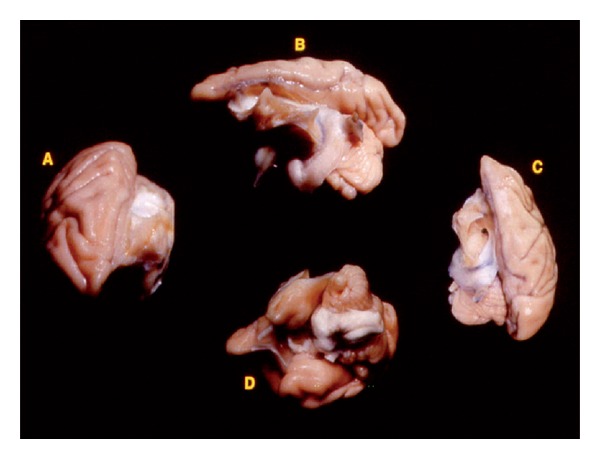
The complete removal of the left hemisphere was performed during infancy. Here, we show the results of the near complete removal of the left hemisphere four years after the initial surgery in the axial plane (A and C), lateral view (B), and orbital view (D).

Using previously described surgical procedures [14, 15], a craniotomy was performed under deep general anesthesia and the left hemisphere was gently retracted from the midline and separated from the diencephalon using a suction pipette (Figure 1). After surgery, all monkeys received postoperative injections of antibiotics for a period of 10 days, and the infant monkeys were returned to their mother until the age of six months. Thereafter, they were housed in a nursery setting for an additional 6 months, at which point they were placed in a social group in a large enriched enclosure (3 m × 2 m × 3 m).

#### 1.2.3. Behavioral Assessment

 In general the subjects appeared to have normal behaviour within their respective social groups with normal peer-to-peer interactions. Feeding behaviour seemed affected with the subjects holding themselves with their left arm and bending down to pick up food with their mouths instead of their right hand. Hemispherectomized subjects were able to groom and had a normal body weight for their respective ages indicating that the removal of the left hemisphere did not affect normal growth or health. The feeding behaviour, however, was indicative of a paresis on the left side. A series of sensory and motor assessments was initiated to test recovery following surgery. Visual assessment was conducted one year following hemispherectomy in both the infant- and adult-lesioned subjects. Thermal sensitivity was assessed in infant-lesioned subjects at three years of age [12, 13]. Motor behaviour was evaluated for three consecutive years after surgery for the infant-lesioned subjects and four years following surgery in the adult-lesioned subjects [14]. As a point of reference, for all behavioural and neural recovery, ipsilateral refers to the left side or that of which the hemisphere was removed; contralateral refers to the right side of the body or that of which the hemisphere remained intact. Behavioural assessments were recorded and analyzed frame-by-frame by two independent observers according to previously published reports [12–14].

## 2. Sensory and Motor Assessment

### 2.1. Vision

Abnormal environmental inputs, during the critical period of development either through sensory deprivation (e.g., eyelid suturing, dark rearing, and enucleation) or cortical injuries lead to dramatic changes at the cellular level and in brain circuitry (reviewed in [16, 17]). The mechanisms underlying visual recovery from large cortical lesions associated with brain plasticity are still unclear and remains an upmost challenge in understanding human patients with lesions restricted to the primary visual cortex (area V1) and those with massive lesions that include all of the visual cortical areas of one cerebral hemisphere (as in hemispherectomy). Animal models suggest that an early unilateral lesion of the visual cortex induces a loss of the contralateral visual field that subsides with time, leading to a complete visual field recovery (see [17]). Neonate hamsters with induced ectopic retinal projections to nonvisual thalamic targets (auditory nucleus and hence auditory cortex) perform as well as normal controls in visual pattern discriminations [18]. In monkeys and humans, the mechanisms underlying recovery of vision in the blind hemifield are not as clear. Only a few cases of spontaneous visual field recovery have been reported in patients born with neonatal malformations of the occipital lobes [19, 20], with recovery attributed to the intact tissue in or surrounding the lesioned area [19, 21] or the contralateral hemisphere [22]. In adults suffering from acquired visual field loss, intensive training through methods such as visual restoration therapy (VRT) can also lead to the reduction of the blind hemifield [23–27]. Here we summarize data collected on basic visual functions as a function of age.

#### 2.1.1. Perimetry

**Figure 2:**
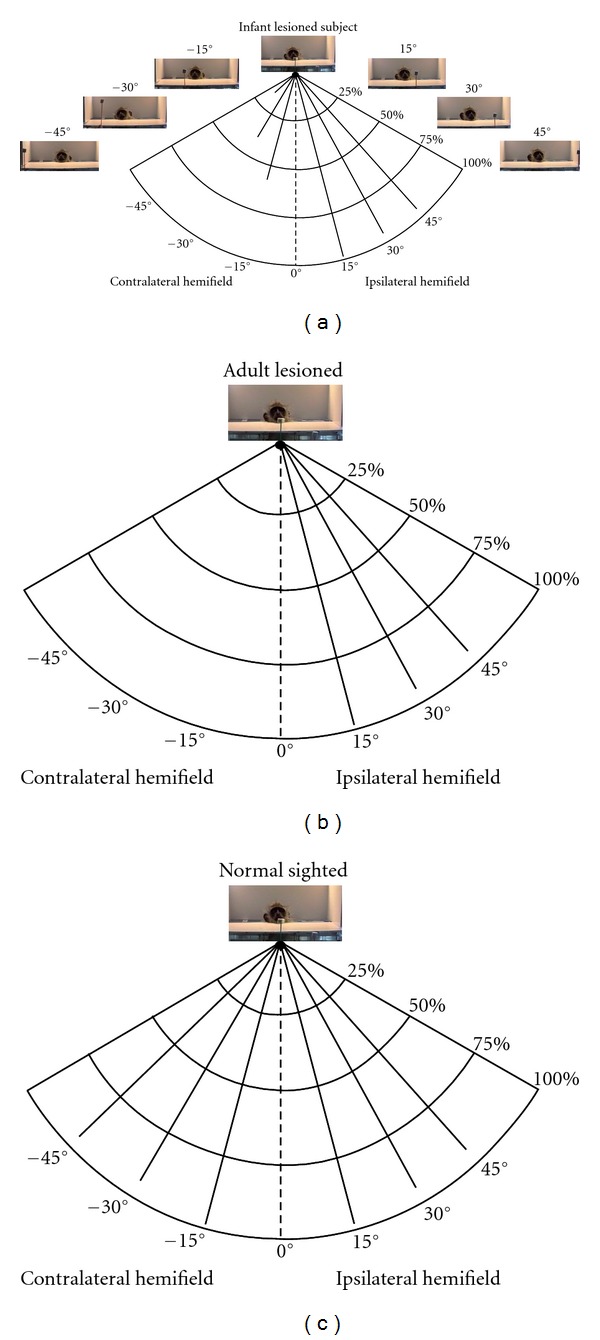
Perimetry: infant-lesioned monkeys (a) were able to detect visual stimuli at 45° in the “blind” hemifield, whereas no visual response could be elicited in the adult-lesioned subjects (b) in the contralateral hemifield. Normal-sighted monkeys had a 75% visual perimetry at 90° in both hemifields (c) (adapted from [13]).

The extent of the visual field for both eyes was measured according to the technique used in the cat [28, 29] and adapted to the monkey [12, 13] one year after the surgery as previously reported [12]. Briefly, the subject was placed in a restraining chair positioned at the center of a perimeter. The monkey was trained to fixate a target (3° of visual angle) at the center of the perimeter positioned at 27 cm from the eyes. A second stimulus (a morsel of fruit on a stick about 1 cm^2^ in size) was then randomly introduced in the visual field at various eccentricities (14 at 15° steps: 0°, 15°, 30°, 45°, 60°, 75°, and 90°, left and right visual fields), and the monkey had to orient its gaze in response to this new stimulus (Figure 2). Visual assessment was performed in three infant monkeys, and the average percent looking behavior is reported here. Adult-lesioned monkeys did not display any type of looking behavior in the blind hemifield in response to visual stimuli. For the control subjects, visual response to stimuli in both hemifields was equal [13].

Orientating responses were apparent in the hemifield contralateral to the lesion in the infant-lesioned subjects. These subjects responded 53%, 35%, and 16% of the time to stimuli presented at 15°, 30°, and 45°, respectively in the blind hemifield (Figure 2(a)). No responses could be elicited beyond 45°. Orientating responses were not seen in the blind hemifield of the adult-lesioned monkeys (Figure 2(b)). In the normal hemifield, occasional errors (e.g., absence of orienting responses to the target) were seen in only the far periphery, a result usually found in normal animals (Figure 2(c)) [13].

#### 2.1.2. Visual Palpebral Reflex and Visual Pursuit

This reflex was tested, with the monkey in a chair, by a sudden thrust of an object toward the open eyes (first at both eyes from the center and then at each eye independently from the sides), and recording if a blink response occurred. Visual pursuit was assessed while an object was slowly moving in the visual field from left to right or right to left. The visual palpebral reflex was always absent in the contralateral visual field for both infant- and adult-lesioned groups. Visual pursuit, when the stimulus started in the intact field, generally stopped at the body midline (i.e., vertical meridian). Conversely, when the stimulus started in the blind field, visual pursuit began only when it reached the midline for both groups of lesioned subjects. For the controls, visual pursuit was smooth in both directions [13].

### 2.2. Motor

Clinical and nonhuman primate data indicate that hemispherectomy results in hemiparesis. Subjects may regain muscle strength in the leg but continue to show weakness in the contralateral arm and hand [5, 30]. In young subjects, motor functions are improved postoperatively, locomotion is preserved, and the hemiplegia of the contralateral limb is improved and replaced by simple voluntary movements [4, 31]. Hemiparetic recovery following hemispherectomy, especially in younger children, has been associated with reorganization of the remaining cortex [32], prior to surgery [11, 33]. Here we describe a series of gross motor tasks to determine the extent of recovery following infant versus adult hemispherectomy.

#### 2.2.1. Open Field

At yearly intervals beginning one year after surgery, infant-lesioned monkeys were assessed for spontaneous gross motor behavior according to previously published methods [14]. Adult-lesioned monkeys were moved into an empty adjoining enclosure 6 months after surgery and assessed individually for spontaneous behavior three years after surgery. Upper and lower limb movements were scored normal for full lift off the ground or abnormal for dragging of the appendage. Limping behavior was also scored as an abnormal movement but was analyzed separately from appendage dragging [14].

**Figure 3:**
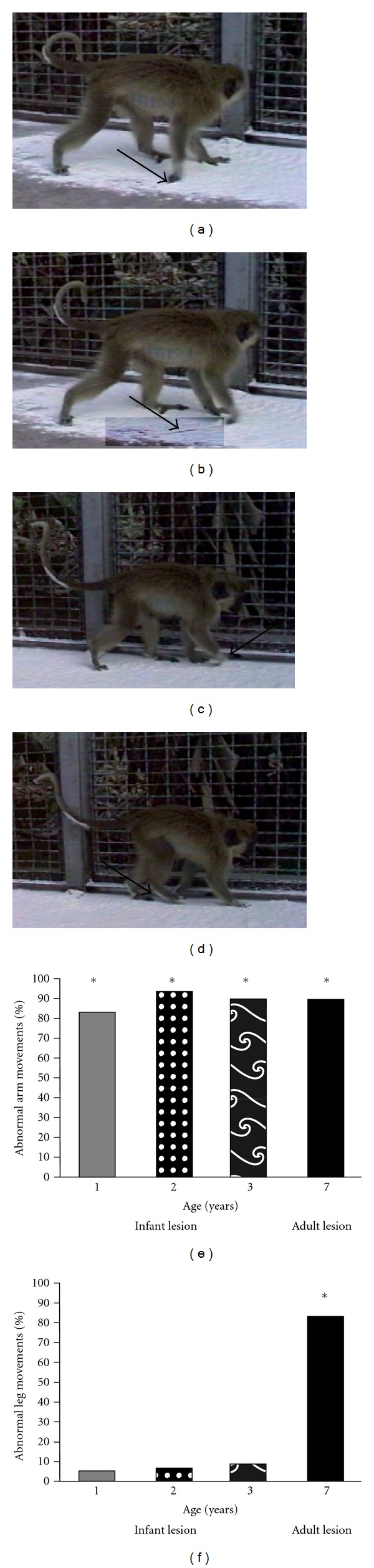
Normal gait was significantly affected by the removal of one hemisphere. The ipsilateral side of the body acts as a control for normal motor movements and did not show any paresis, also referred to as the nonparetic side of the subject. Panels (a)–(d) are indicative of a typical gait sequence. As the forelimb moves forward, the contralateral appendages drag on the ground (arrow panel a). The hand dragging continues through the entire forward motion (arrow panels b and c). The hind limb is fully removed from the ground and does not drag (arrow d). At each time point and lesion group there is a significant effect of the hemispherectomy on upper limb movement defined as arm drags/total forward arm movements (e). A number of abnormal leg movements (drags and limps) as a percentage of total leg movements was significantly elevated over the expected rate of zero in the adult-lesioned group only (f). Years 1, 2, and 3 refer to the age of the infant-lesioned subjects at testing which also corresponds to the years after lesion. For the adult-lesioned subjects, testing occurred 3 years after initial surgery when the subjects were 7 years old. *P < 0.001 adapted from [14].

Normal gait was observed on the ipsilateral side with the appendages clearly being lifted off the ground as the limbs moved forward for both age groups at each observation period. Both infant- and adult-lesioned subjects had pervasive upper limb dysfunction with 90% of total arm movements resulting in the hand being dragged along the ground (Figure 3). Lower limb movement difficulties were less frequently observed in infant-lesioned subjects with less than 10% of total movements resulting in dragging. The lower limb in the adult-lesioned subjects was also less affected with 16% of the leg movements being accompanied with a foot drag. However, when taking into account limping as an abnormal movement, the lower limb in the adult-lesioned monkeys was significantly affected. This suggests that lower limb recovery in the young-lesioned animals is much stronger than the upper limb that remains hemiparetic [14].

#### 2.2.2. Horizontal Bar Crossing

 The vervet monkey is an agile species with the ability to cross a horizontal bar that is a complex visuo-spatial-motor task, from a very young age.At yearly intervals, infant-lesioned subjects were assessed for their ability to perform bar crossing. Adult-lesioned subjects were moved into an empty adjoining enclosure 6 months after surgery and assessed individually for spontaneously crossing the bar. Behavior was scored as follows: (1) attempt to grab the bar with the contralateral fore- or hind-limbs was scored either as a successful or unsuccessful grab, with the successful attempt being defined as the contralateral appendage fully grasping the bar and (2) no attempt to grab the bar while walking across the horizontal bar defined as a movement across the bar using only the ipsilateral limbs/appendages. Video was analyzed frame-by-frame for each time point and group (see Burke et al., for more details [14]).

**Figure 4:**
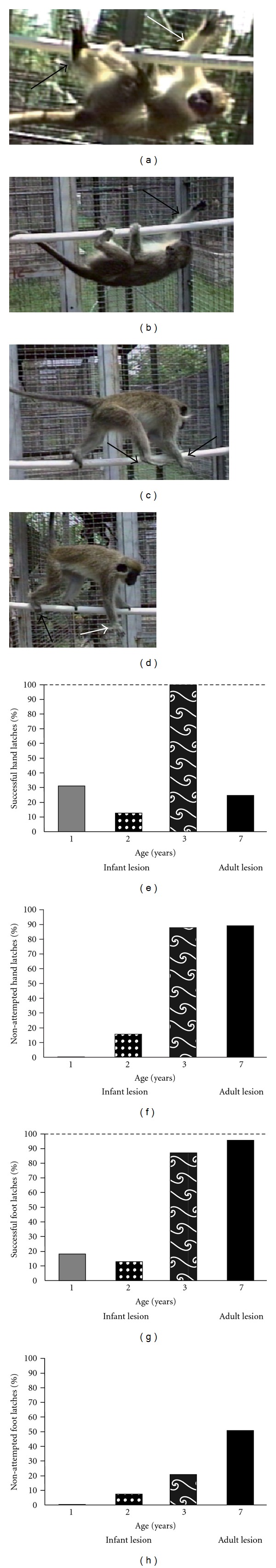
At a young age, the subjects made a significant number of unsuccessful attempts to grab the horizontal bar with their hands and would transverse the bar upside down (a and e). The subjects would typically overreach the bar with their arms and glide their arms across the bar until their hands were able to latch on (white arrows in a and b). The lower limb also had difficulty latching onto the bar during the first two postoperative years after the surgery (black arrow in panels a and g). At two years of age, the monkeys began not to attempt to use the contralateral upper limb to transverse the bar (black arrows in panel c). Typically the subject would successfully use the affected hind limb (black arrow in panels d and h) and would not attempt to use the front limb (white arrow in panels d and f) to transverse the horizontal bar. By 2 years after surgery the subjects were able to walk upright across the bar. Dashed line (e and g) represents the expected value for a normal monkey. % successful was determined as successful latches (hand or foot)/total attempts to latch onto the horizontal bar. The ipsilateral side of the body acts as an internal control for normal motor movements and did not show any paretic movements. Years 1, 2, and 3 refer to the age of the infant-lesioned subjects at testing which also corresponds to the years after lesion. For the adult-lesioned subjects, year 4 refers to the number of years after initial surgery. Adapted from [14].

The infant-lesioned subjects were unable to transverse the bar by walking upright as a normal monkey for the first 2 years following surgery; instead, they would transverse the bar hanging upside down from the bar. The subjects would hang onto the bar with the ipsilateral limbs, overreach the bar with the contralateral limb such that the entire arm or leg was completely extended above the bar, and then glide the limb down the bar until either the hand or foot was able to latch onto the bar (Figure 4). The monkeys were typically unable to transverse the entire length of the bar without falling to the ground. By the third year, the monkeys started to use a new strategy for crossing the horizontal bar by not attempting to use the contralateral limb. When the monkeys attempted to use the contralateral arm on the horizontal bar, they displayed a 100% success rate. However, 88% of the movements across the bar did not involve the contralateral hand. The subjects did attempt to use the contralateral leg 79% of the time and had an 88% success rate when the leg was used. The adult-lesioned subjects were able to successfully walk upright the entire length of the horizontal bar. When the monkeys attempted to use the contralateral arm on the horizontal bar, they displayed a 25% success rate; however, the vast majority of movements across the bar did not involve the contralateral hand [14].

#### 2.2.3. Turning Behavior

During open field motor assessment, turning preference was scored as a 90° or 180° turn ipsi- or contralateral to the lesioned side of the body. An average of 172 ± 43 turning behaviors were scored, and at each time point a consistent ipsilateral turning preference was recorded. At 1 year of age, 70% of turns were ipsilateral. This increased to 86% and 89% at the age of 2 and 3, respectively, whereas the adult-lesioned animals had a 94% ipsilateral turning preference. There were no differences in turning behavior between age groups, or infant- versus adult-lesioned subjects indicating that this function never recovered over time, with the animals still showing a marked preference for the nonblind hemifield [14].

### 2.3. Temperature Sensitivity

Previous studies have shown that hemispherectomy patients are able to perceive tactile stimulation applied to their paretic leg. Functional magnetic resonance imaging (fMRI) studies further showed that this type of stimulation leads to activation of the intact primary and secondary somatosensory cortices, suggesting the existence of ipsilateral, nondecussating pathways from the periphery [34, 35]. Here we measured thermal sensitivity of the upper and lower limbs as a measure of residual somatosensation in infant-lesioned subjects.

**Figure 5:**
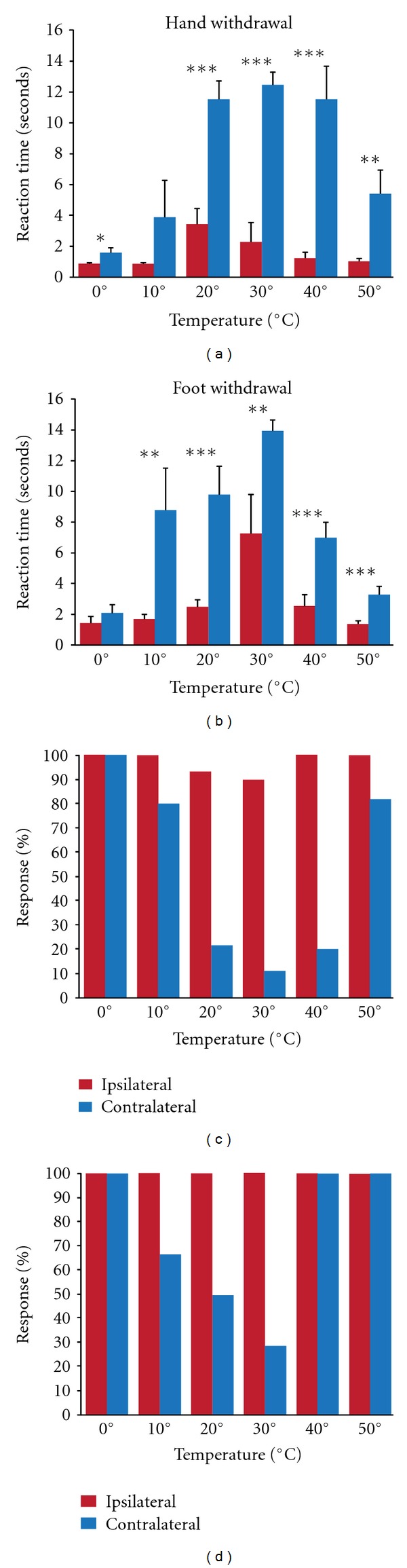
Thermal sensitivity: thermal sensitivity was tested in the infant-lesioned subjects at 3 years of age. The ipsilateral side of the body acts as a control for normal response to thermal stimuli and, as stated earlier, did not show any paretic movements. Withdrawal times were significantly longer for the contralateral (paretic side) than for the ipsilateral (nonparetic) limb (a and b). The contralateral lower limb tended to have higher response rates than upper limb to thermal sensitivity (c and d). Withdrawal response rates did not differ between upper and lower ipsilateral limbs. *P < 0.05, **P < 0.01, ***P < 0.001.

The subjects were placed in a restraining chair with their arms and legs freely moving. The subjects' fingers or toes were randomly immersed into recipients containing water at varying degrees of temperature (0, 10, 20, 30, 40, and 50°C). Whereas the 30°C can be considered as a neutral temperature, the 20 and 40°C are innocuous cold and warm stimuli, respectively. The 10 and 0°C temperatures fall within the noxious cold range whereas the 50°C is a noxious heat stimulus. If the monkey did not withdraw the appendage from the water within 16 seconds, the trial was terminated and counted as a nonresponse (Figures 5(c) and 5(d)). Two years after the surgery both upper (fingers) and lower (toes) limbs (ipsi- and contra-) were tested at each temperature, and the withdrawal reaction time was recorded via frame-by-frame analysis of the video (Figure 5). Appendage withdrawal responses were analyzed in three monkeys, and time of withdrawal was compared by *t*-test between ipsi- and contralateral appendages.

In line with our expectations, withdrawal times of the ipsilateral upper and lower limb for the neutral and innocuous temperatures were longer than for the noxious stimuli (Figure 5). Whereas the animal withdrew the limb in less than 30% of the cases for the neutral temperatures, this increased to 100% for the painful cold and heat stimuli. For the ipsilateral lower limb, the average withdrawal times for neutral, innocuous warm and noxious heat stimuli were 7.33 ± 2.49, 2.57 ± 0.72, and 1.36 ± 0.2, seconds respectively, (Figure 5). A similar response pattern was observed for the contralateral stimuli with this noticeable difference that withdrawal times were significantly longer for all tested temperatures. The percentage of withdrawal responses to noxious cold and heat stimuli remained close to 100% for the contralateral limb. For the contralateral upper limb, average withdrawal times and percentage of withdrawals did not differ for the neutral and innocuous temperatures. However, for the lower limb, withdrawal times were shorter for the innocuous compared to the neutral temperatures. For both the upper and lower limb, withdrawal times for the noxious temperatures were shorter than for the innocuous temperatures. Together, these data suggest that while the contralateral limbs retained thermal sensitivity, there was a clear impairment in the perception of the innocuous warm and cold temperatures, especially for the upper limb.

## 3. Discussion

The ability of the cerebral cortex to adapt and reorganize following insult early in life is remarkable but has been difficult to study systematically in human infants. Data has been mostly accumulated from case studies and suggest that lesions sustained in early childhood lead to better recovery [36] indicating a prominent anatomical reorganization in human subjects [37–39]. Here we present a 20-year culmination of data on our nonhuman primate model of early-life hemispherectomy. For individuals with medically required hemispherectomy, it is possible that neural reorganization had already begun much before surgery but was masked by the dysfunctional hemisphere. The release from the “negative” influence exerted by the diseased hemisphere would give the appearance of a rapid reorganization of the remaining hemisphere [10]. Data from our laboratory as well as those from human studies suggest a critical role for subcortical and brainstem structures as well as corticospinal tracts in the neuroanatomical reorganization which result in the remarkable behavioral recovery following hemispherectomy [12, 40–43].

### 3.1. Summary and Clinical Parallels

#### 3.1.1. Vision

Clinical studies involving individuals that underwent hemispherectomy conclude that there is marked improvement of life post-surgery, mainly due to the cessation of seizure activity [5, 6, 44, 45]. Recovery largely depends on the system in question. We report here that infant-lesioned monkeys had residual vision up to 45° in the blind hemifield compared to adult-lesioned subjects, where a behavioral response could not be elicited in the blind hemifield. We have previously reported that these subjects demonstrate a pervasive ipsilateral turning preference that may be indicative of a visuospatial impairment related to hemianopia. A preference for processing visual information from the ipsilateral hemifield (i.e., the unaffected field of vision) may be responsible for ipsiversive turning [14]. These residual visual capabilities parallel clinical findings for individuals who underwent hemispherectomy for treatment of intractable epilepsy. Indeed, in human hemispherectomy patients, nonreflexive visual responses can be elicited in the blind hemifield [46]. Responses to visual stimuli presented in the hemianopic side have been classified according to the patient's implicit (Type I blindsight) or explicit (Type II blindsight) acknowledgement of the presence of the stimulus [7]. The ability of infant-lesioned subjects in the current study to actively orientate towards food rewards in the blind hemifield is reminiscent of Type II blindsight. The lack of visual orientation in the blind hemifield of the adult-lesioned subjects suggests that like recovery in sensorimotor abilities, the younger cortical lesioned subjects tend to recover function more completely than their adult-lesioned counterparts [14]. Results from lesions restricted to the striate cortex of cats and monkeys [47] support a relationship between the degree of visual recovery and the age at which the lesion was performed [48, 49].

In clinics, it is generally accepted that early-lesioned patients have a larger chance of residual vision [50]. A recent clinical study suggests that children who have unilateral or bilateral loss of their occipital lobe(s) are capable of retaining normal vision in both visual fields when tested with a forced-choice preferential-looking perimetric method. A child who underwent a complete hemispherectomy at the age of 59 months could consciously detect light throughout the visual field. This is in sharp contrast with a visual perimetry of only 30° in a patient who underwent hemispherectomy at 13 years of age, suggesting that visual recovery is dependent upon age of lesion. In both patients, the luminance of the visual target had to be at least 45 cd/m^2^ to allow detection in the affected visual hemifield, compared to 5 cd/m^2^ in the unaffected hemifield [36]. Moreover, a patient born with developmental anomaly of both occipital lobes demonstrated a large reduction of the bilateral scotomas leading to an expansion of the total visual field [19]. Residual vision varies among patients and may not be entirely dependent on the age of surgery since residual vision has also been reported in patients who underwent hemispherectomy during late adolescence and early adulthood [7, 51, 52].

In patients and monkeys with massive unilateral lesions of the primary visual cortex, residual vision in the blind hemifield (Type I and Type II blindsight) has been ascribed to ipsilateral extrastriate cortical areas that receive inputs via the colliculopulvinar pathway [53, 54]. In the case of anatomical hemispherectomy where all visual cortical regions of one hemisphere have been removed, residual vision is dependent upon the contribution of the remaining hemisphere as demonstrated by brain-imaging methods. Thus, it was shown that visual stimulation of the blind hemifield in hemispherectomy patients activates visual cortical areas in the remaining hemisphere [55, 56]. A more recent, DTI study by Leh and colleagues [52] highlighted the pathway by which the visual information presented to the blind field reaches the contralateral visual cortex. Indeed, these authors traced a retinofugal projection to the SC ipsilateral to the removed hemisphere that reaches the contralateral SC, then the pulvinar and the extrastriate cortices [7, 52]. This indicates that the SC ipsilateral to the removed hemisphere survives the hemispherectomy and can be activated by visual stimuli presented in the hemianopic field.

**Figure 6:**
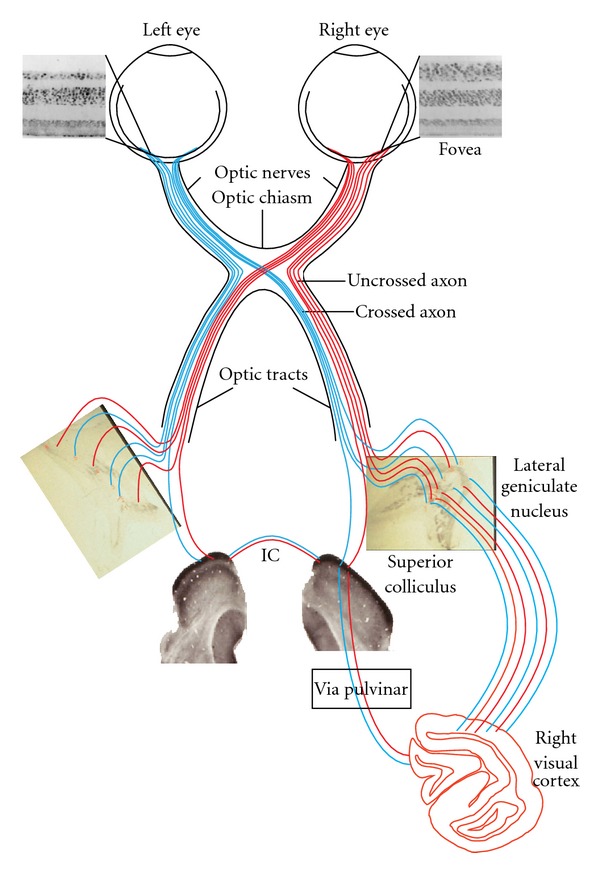
Suggested anatomical pathway for visual field recovery following early hemispherectomy (based on a series of anatomical studies performed on the same monkeys). The deafferented left temporal hemiretina (T) still contains ganglion cells [57] that send their axons to the remaining left dLGN as a dead end [12]. Retinofugal projections to the left superior colliculus (SC) are still robust and maintain a normal metabolic rate as measured by cytochrome oxydase immunochemistry [59]. The information reaching the left SC is transferred to the right SC via the intertectal commissure (IC), the right Pulvinar (P), and the occipital cortex of the remaining right hemisphere. The deafferented right nasal hemiretina (N) sends crossed projection through the optic chiasm (OC) to the appropriate layers of the remnants of the left dLGN and to the left SC [15, 58, 59, 62]. The left Pulvinar is severely atrophied and receives no retinal projections. Projections from the afferented portions of the retinae (left nasal and right temporal) reach their subcortical targets in a normal fashion en route to the occipital cortex of the right hemisphere.

Anatomical evidence, from our laboratory on the same hemispherectomized monkeys used in our behavioral experiments, suggests that retinal and subcortical visual structures survive the lesion but to varying degrees [12]. Although there is a massive transneuronal degeneration of retinal ganglion cells in the fovea, the peripheral retina remains unaffected [57]. The main thalamic target of the retina, the dorsolateral geniculate nucleus (dLGN), ipsilateral to the removed cortex undergoes a major loss of neurons and an intense gliosis [58]. Notwithstanding a large volume reduction, the dLGN still receives projections from each retina ending in the appropriate layers [15]. The paucity of surviving neurons within the magno- and parvocellular layers does not make the dLGN a likely candidate for sustaining residual vision. On the other hand, the SC retains functional capabilities as revealed by cytochrome oxidase activity and receives normal retinal inputs. Unlike the dLGN, the ipsilateral colliculus undergoes only moderate neuronal reduction following hemispherectomy and remains mostly intact [59]. Moreover, the ipsilateral substantia nigra also remains intact with no obvious volume or neuronal loss [60]. The substantia nigra plays an important role in saccadic eye movements with the lateral part committing the majority of its projections to the nigrotectal pathway [61]. As suggested by tractography studies in human hemispherectomy patients, it seems, therefore, that the collicular system and the lateral substantia nigra play an important role in residual visual capabilities.  Figure 6 summarizes the neural substrates implicated in residual vision in the blind hemifield of hemispherectomized monkeys.

#### 3.1.2. Motor and Somatosensory Functions

Our results on the behavioral recovery of sensory-motor behaviors in the infant hemispherectomized monkey parallel to those reported in clinical cases [5, 30, 63] as well as those reported in neonatal feline models of hemispherectomy [64–66]. The infant-lesioned monkeys tend to regain function in the lower limb within a year after surgery, but the upper limb appears to remain hemiparetic, mirroring clinical data where subjects may regain muscle strength in the leg but continue to show weakness in the contralateral arm and hand with an intact tactile detection [5, 30, 35]. The ineptitude of our subjects to effectively transverse the horizontal bar reflects an inability to integrate complex visuospatial, somatosensory, and motor information. A diminished tactile sensation became especially apparent with the overreaching and gliding of the arm and leg until the subject was able to latch onto the bar.

All the preserved functions, motor as well as somatosensory, following hemispherectomy in both humans and nonhuman primates have been attributed either to an extensive anatomical reorganization or to the use of compensatory mechanisms involving either the remaining cortex or subcortical residual structures. There is evidence that ipsilateral projections may play a role in the retention of function following hemispherectomy. Patients are able to perceive tactile stimulation applied to their paretic leg, and fMRI studies have shown that this type of stimulation leads to activation of the intact primary and secondary somatosensory cortices, suggesting ipsilateral pathways from the periphery [34, 38, 39, 67]. In one particular patient, thermal sensitivity was impaired on the paretic side of the body, but the patient was able to discriminate temperature changes on the skin. This patient experienced a pricking-burning sensation of the skin (allodynia) that was exacerbated by cold. fMRI data indicated that the allodynia experienced on the paretic limbs was processed in the remaining cortex in areas normally associated with pain processing [67]. The remaining cortex has also been shown to undergo significant reshaping in the motor and sensorimotor cortical representations [37, 68–71]. There is evidence from fMRI studies that physical training with the paretic lower limb results in cortical activation of the remaining primary sensorimotor, supplementary motor, cingulate, and secondary somatosensory cortices, suggesting an experiential or active-dependent recovery [72].

**Figure 7:**
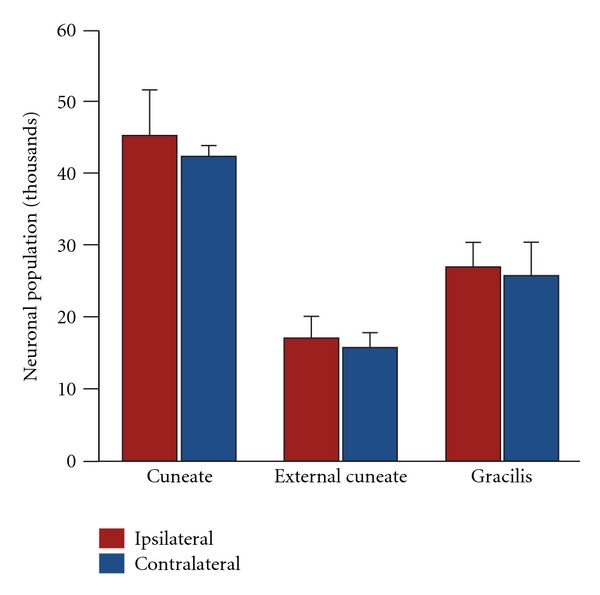
Design-based stereology neuronal counts in the dorsal column nuclei. There were no neuronal population differences between ipsi- and contralateral subdivisions of the dorsal column nuclei.

The ability of the remaining hemisphere to assume functional control over the ipsilateral body may be due to a reorganization of the brainstem tracts such as the corticospinal tract and medial lemniscus [41–43, 72]. In a child with intractable epilepsy in the left hemisphere, a presurgical fMRI study showed activation of the right, but not of the left primary motor cortex following tactile and motor stimulation of the right hand. These results suggest that reorganization occurred prior to surgery and that the corticospinal fibers originating from the nonaffected hemisphere mediated the reorganization [41]. In normally developing children, ipsilateral corticospinal connections remain intact until around 10 years of age [73] providing an anatomical substrate for reorganization following early-life dysfunction. Diffusion tensor imaging (DTI) data further suggest that the medial lemniscus may play a vital role in sensory recovery as it retains symmetry following early-life hemispherectomy [43]. Using design-based stereology, our group has reported relatively preserved contralateral dorsal column nuclei (gracilis, cuneatus, and external cuneatus) following hemispherectomy in infant primates (Figure 7) that could, in part, mediate the behavioral recovery [40].

The differential effect of hemispherectomy on the upper- versus lower limbs may also be mediated by networks of interneurons within the spinal cord [5, 30, 42, 74]. In human and nonhuman primates, lower-limb locomotion is thought to be under the control of neuronal circuits of the central pattern generator, within the spinal cord, whereas the upper limbs are under the control of corticospinal pathways [5, 74]. It has also been suggested that early cortical lesions lead to a reinforcement of the ipsilateral corticospinal tracts, whereas for cortical lesions sustained late in life recovery may be mediated by the cortico reticulospinal pathway [42].

### 3.2. Conclusion

The data presented here suggest that significant functional recovery occurs after the removal of one hemisphere in monkeys with no preexisting neurological dysfunctions. The nonhuman primate model presented here offers a unique opportunity for studying the behavioral and functional neuroanatomical reorganization that underlies developmental plasticity. All the preserved visual, motor, and sensory functions following hemispherectomy in both humans and nonhuman primates have been attributed to an extensive anatomical reorganization or to the use of compensatory mechanisms involving either the remaining contralateral cortex or subcortical residual structures [12]. The anatomical state of the visual system of infant-lesioned monkeys adds support to the implication of the collicular system in mediating the sparing of vision in the contralateral hemifield observed in hemispherectomized humans [7, 50]. Anatomical and imaging studies further suggest that recovery of the motor and somatosensory (both tactile and thermal) functions may be subserved by existing, nondegenerating, ipsilateral projections of the medial lemniscus, corticospinal tract, and dorsal column nuclei as well as preserved superior colliculi and substantia nigra [60]. The extent and manner to which subcortical areas, the brainstem, and the spinal cord participate in functional reorganization following early-life hemispherectomy remains unresolved. The nonhuman primate model presented here has provided a significant contribution to our understanding of the behavioral and functional neuroanatomical reorganization that underlies developmental plasticity.
